# Comparing machine and deep learning‐based algorithms for prediction of clinical improvement in psychosis with functional magnetic resonance imaging

**DOI:** 10.1002/hbm.25286

**Published:** 2020-11-13

**Authors:** Jason Smucny, Ian Davidson, Cameron S. Carter

**Affiliations:** ^1^ Department of Psychiatry University of California Davis California USA; ^2^ Department of Computer Science University of California Davis California USA

**Keywords:** cognitive control, frontoparietal, neuroimaging, prognosis, schizophrenia

## Abstract

Previous work using logistic regression suggests that cognitive control‐related frontoparietal activation in early psychosis can predict symptomatic improvement after 1 year of coordinated specialty care with 66% accuracy. Here, we evaluated the ability of six machine learning (ML) algorithms and deep learning (DL) to predict “Improver” status (>20% improvement on Brief Psychiatric Rating Scale [BPRS] total score at 1‐year follow‐up vs. baseline) and continuous change in BPRS score using the same functional magnetic resonance imaging‐based features (frontoparietal activations during the AX‐continuous performance task) in the same sample (individuals with either schizophrenia (*n =* 65, 49M/16F, mean age 20.8 years) or Type I bipolar disorder (*n* = 17, 9M/8F, mean age 21.6 years)). 138 healthy controls were included as a reference group. “Shallow” ML methods included Naive Bayes, support vector machine, K Star, AdaBoost, J48 decision tree, and random forest. DL included an explainable artificial intelligence (XAI) procedure for understanding results. The best overall performances (70% accuracy for the binary outcome and root mean square error = 9.47 for the continuous outcome) were achieved using DL. XAI revealed left DLPFC activation was the strongest feature used to make binary classification decisions, with a classification activation threshold (adjusted beta = .017) intermediate to the healthy control mean (adjusted beta = .15, 95% CI = −0.02 to 0.31) and patient mean (adjusted beta = −.13, 95% CI = −0.37 to 0.11). Our results suggest DL is more powerful than shallow ML methods for predicting symptomatic improvement. The left DLPFC may be a functional target for future biomarker development as its activation was particularly important for predicting improvement.

## Graphical abstract

The performance of shallow machine learning algorithms was compared to deep learning when predicting symptomatic improvement in early psychosis using frontoparietal activation data from a cognitive control task. Deep learning outperformed all shallow algorithms for both binary and continuous classifiers. Explainable artificial intelligence (XAI) methods suggested the deep learner primarily used left dorsolateral prefrontal cortex activation to predict clinical improvement, providing an example of how XAI can be used to "demystify" the "black box" problem inherent in all machine learning algorithms.

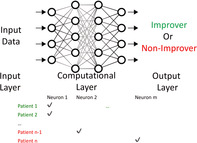

## 1 INTRODUCTION

Response to treatment in psychotic disorders is highly variable, and biomarkers of treatment response have been lacking to date. As a result, trial and error remains the basis for care in early psychosis, and poor outcomes continue to be common in individuals even when duration of untreated psychosis is relatively short and specialized clinical care is provided. Early identification of patients who are less likely to have good treatment responses would help identify individuals who would benefit from the early introduction of alternative and/or supplemental interventions.

To that end, we have previously demonstrated that functional magnetic resonance imaging (fMRI)‐based measures of cognitive control‐related brain activity collected during initial engagement in treatment predict symptomatic “improvement” (defined as showing at least 20% improvement in total Brief Psychiatric Rating Scale [BPRS] score after 1 year) in early psychosis patients after 1 year with 66% accuracy using logistic regression (Smucny, Lesh, & Carter, 2019). Although this finding was an important step toward developing a neuroimaging‐based prognostic regimen, to be clinically useful a predictive battery should display at least 80% accuracy.

As logistic regression classifies based on a logarithmic function, its accuracy is limited by the fit of that function to the data. Furthermore, logistic regression‐based classification is only able to utilize the features/independent variables/descriptors provided. Machine learning (ML) classifiers utilize more complex functions that may improve performance, although most of these classifiers are “shallow” and therefore again limited to using the features provided. Deep (machine) learning (DL) classifiers, however, have more compositional capabilities to create new features from the features provided by utilizing a layered network, potentially allowing for more accurate representation of the data and thus more precise mapping (Durstewitz, Koppe, & Meyer‐Lindenberg, 2019; Montufar, Pascanu, Cho, & Bengio, 2014). Consider a simple illustrative example with two input features—brain activations from “Region 1” and “Region 2.” Shallow ML methods will find a classification function solely using activation values from these regions as parameters. DL methods, on the other hand, will try many different combinations of them and learn which combination is the most predictive, in a process referred to as end‐to‐end learning. Furthermore, explainable artificial intelligence (XAI) methods have been recently developed (Davidson, Gourru, & Ravi, 2018; Sambaturu et al., 2020) that can extract which feature(s) most strongly influenced the deep learner's classification decisions, helping to demystify the “black box” problem inherent in ML methods. As DL is combinatorial, these “rules” for classification may be complex. In the example with two brain regions, XAI may find rules such as (A) “If activation >0.5 in Region 1 classify as B,” (B) “If activation <0.5 in Region 1 and >0.5 in Region 2, classify as “B,” (C) otherwise classify as “A.”

Here, we evaluate the performance of six commonly used ML methods as well as a deep learner in predicting symptomatic improvement in early psychosis individuals after 1 year of treatment. The shallow ML methods vary in their complexity and approach. We were particularly interested in determining if the ability of DL to combine features into more complex predictors may show performance enhancement versus shallow architectures and our previous logistic regression‐based finding. In order to compare classification accuracies, therefore, here we analyzed the same dataset and frontoparietal features that were used in our previous study using logistic regression. In alignment with previous findings (Krizhevsky, Sutskever, & Hinton, 2012; Le et al., 2012), we hypothesized the best performance would be observed using the deep learner.

## 2 METHODS

### 2.1 Sample

As described previously (Smucny et al., 2019), complete (baseline and follow‐up) neuroimaging AX‐CPT data were available for 171 individuals (139 with schizophrenia [SZ], 32 with Type I bipolar disorder [BD] with psychotic features). Of this sample, follow‐up clinical data were available for 82 individuals (65 SZ, 17 BD). 138 healthy controls (HCs) were included to verify the task was activating expected frontoparietal regions (see Section 2.4) and as a reference group. Neuroimaging AX‐CPT data from the 82 patients with complete (baseline and follow‐up) datasets have been used in previous studies as follows: (Lesh et al., 2013)—53 controls and 18 patients, (Lesh et al., 2015)—34 HC and 20 psychosis, (Niendam et al., 2014)—23 HC and 11 psychosis, (Smucny et al., 2018)—52 HC and 43 psychosis, (Yoon et al., 2008)—21 HC and 6 psychosis. Individuals were recruited as outpatients from the University of California, Davis (UCD) Early Diagnosis and Preventive Treatment (of Psychosis) (EDAPT) research clinic (http://earlypsychosis.ucdavis.edu). Treatment in the clinic follows a coordinated specialty care for early psychosis model delivered by an interdisciplinary treatment team. Treatment includes detailed clinical assessments using gold‐standard structured clinical interviews and medical evaluations, targeted pharmacological treatments including low dose atypical antipsychotic treatment, individual and family‐based psychosocial education and support, cognitive behavioral therapy for psychosis, and support for education and employment. The Structured Clinical Interview for DSM‐IV‐TR (First, Spitzer, Gibbon, & Williams, 2002) was used for diagnosis of psychopathology. Diagnoses were confirmed by a group of trained clinicians during case conferences. All patients reported psychosis onset within 2 years of the date of informed consent. Patients were excluded for a diagnosis of major medical or neurological illness, head trauma, substance abuse in the previous 3 months (as well as a positive urinalysis on the day of scanning), Weschler Abbreviated Scale of Intelligence‐2 score (Weschler, 1999) score < 70, and magnetic resonance imaging (MRI) exclusion criteria (e.g., claustrophobia, metal in the body). Control participants were excluded for all of the above as well as a history of Axis I mental illness or first‐degree family history of psychosis. All participants provided written informed consent and were compensated for participation. The UCD Institutional Review Board approved the study. Medication regimen (type and dose) was assessed by clinical records at baseline and follow‐up. Medication compliance was based on self‐report. Medicated patients at follow‐up all self‐reported at least medium compliance with antipsychotic medication during the treatment period (except for two SZ individuals who were missing compliance data at follow‐up). Symptoms were assessed using the 24‐point BPRS (Ventura et al., 1993) rescaled to a lowest score of zero (i.e., score of 24 = score of 0). At baseline, all patients had BPRS scores > = 5 to ensure sufficient resolution to detect a 20% improvement in score at follow‐up.

### 2.2 Task description

The AX‐CPT and associated task parameters have been described in detail elsewhere (Braver, Paxton, Locke, & Barch, 2009; Cohen, Barch, Carter, & Servan‐Schreiber, 1999; Henderson et al., 2012; Lesh et al., 2013; Phillips, Salo, & Carter, 2015). Briefly, participants are presented with a series of cues and probes and are instructed to make a target response (pressing a button with the index finger) to the probe letter “X” only if it was preceded by the cue letter “A.” All cues and nontarget probes require nontarget responses (pressing a button with the middle finger). Target sequence trials (i.e., “AX” trials) are frequent (60–70% occurrence) and set up a prepotent tendency to make a target response when the probe letter X occurs. As a result, a nontarget sequence trial in which any non‐A cue (collectively called “B” cues) is presented and followed by a probe letter X (i.e., “BX” trials) requires proactive cognitive control (e.g., maintenance of the inhibitory rule over the delay time) (Braver et al., 2009). Consistent with prior work (Henderson et al., 2012), individual subject data was only included in analyses if results suggested the subject understood the AX‐CPT (specifically, accuracy greater than 44% on AX trials and 50% on BY trials at both baseline and follow‐up). Participants were combined across two task protocols collected from two MRI scanners. Parameters for each protocol (AX‐1 and AX‐2) are provided in Supplementary Table 1a. The task was presented using EPrime2 software (Psychology Software Tools, Inc.). The behavioral index of proactive cognitive control was d‐prime context, a function of AX hits minus BX false alarms (Cohen et al., 1999).

### 2.3 fMRI scanning parameters and preprocessing

Functional images were acquired with a gradient‐echo T2* blood oxygenation level‐dependent (BOLD) contrast technique as outlined in Supplementary Table 1b. AX‐1 was performed in a 1.5 T scanner (GE Healthcare), and AX‐2 in a 3.0 T scanner (Siemens).

fMRI data were preprocessed using SPM8 (Wellcome Department of Imaging Neuroscience, London). Briefly, images were slice‐timing corrected, realigned, normalized to the Montreal Neurological Institute template using a rigid‐body transformation followed by nonlinear warping, and smoothed with an 8 mm full‐width‐half‐maximum Gaussian kernel. All individual fMRI runs had less than 4 mm of translational within‐run movement, 3° of rotational within‐run movement, and 0.45 mm of average framewise displacement (calculated using the fsl_motion_outliers tool) (https://fsl.fmrib.ox.ac.uk/fsl/fslwiki/FSLMotionOutliers). Mean displacement did not differ between Improvers and non‐Improvers (*t* = 1.42, *p* = .16). All participants had at least two fMRI runs surviving these criteria. Preprocessing pipelines were identical for AX‐1 and 2.

### 2.4 fMRI analysis and prespecified ROI selection

First‐level effects were modeled with a double‐gamma function with temporal derivatives using the general linear model in SPM8. Rigid‐body motion parameters were included as single‐subject regressors in order to partially account for movement effects. B > A cue (correct trials only) contrast images (parameter estimates) were generated for each subject. The B > A cue contrast measures response under conditions of high versus low proactive cognitive control (Lesh et al., 2013; Lesh et al., 2015). All trial types were modeled (AX/AY/BX/BY) and only correct responses were used to create first‐level images, consistent with previous studies (Lesh et al., 2013; Lesh et al., 2015). Whole‐brain analyses across the final sample (HCs and patients with follow‐up data) using the B > A contrast were used to confirm significant (height threshold *p* < .001, cluster threshold *p* < .05 [whole brain FDR‐corrected]) activation in expected brain regions (bilateral DLPFC/SPC) for both protocol versions (AX‐1 and AX‐2).

For ML using first‐level images and as extracted in our previous logistic regression study (Smucny et al., 2019), BOLD response was extracted from prespecified bilateral, 5 mm radius spherical DLPFC and SPC ROIs. Although this size was chosen arbitrarily, previous work from our group suggests varying ROI radius between 4 and 8 mm does not substantially affect AX‐CPT task‐associated response patterns in psychosis (Smucny et al., 2018). The DLPFC ROI was taken from a previous study from an independent dataset (MacDonald III, Cohen, Stenger, & Carter, 2000). The SPC ROIs was taken from a meta‐analysis of executive function in SZ (Minzenberg, Laird, Thelen, Carter, & Glahn, 2009). Mean task‐associated activation from these ROIs was extracted using the Marsbar toolbox (Brett, Anton, Valabregue, & Poline, 2002). Activations were adjusted for differences in protocol version prior to further analysis by calculating standardized residuals from the linear regression of protocol version by each measure. The adjusted values from each of the four ROIs were then utilized as input features (i.e., four total inputs) for ML/DL. Unlike our previous study, AX‐CPT performance was not used as a predictor as our previous logistic regression study found no association between behavior and outcome. As a supplementary analysis, we also extracted voxelwise data within these ROIs for use as features, adjusted for protocol version as described above.

### 2.5 Machine learning

“Shallow” and DL algorithms were trained to predict “Improver” (vs. non‐Improver) status, defined as >20% decrease in Total BPRS score from baseline rescaled to a lowest score of zero (Howes et al., 2017), as well as change in Total BPRS score.

Shallow algorithms were employed using Weka software (University of Waikato, New Zealand) and included Naive Bayes, support vector machine (SVM), K Star (K *), AdaBoost, J48 decision tree, and random forest. Classifier accuracies were calculated by averaging performance across 1,000 random assortments of 90% training data and 10% test data for each algorithm.

### 2.6 Naive Bayes

Naive Bayes (Maron, 1961) compares the probability of observing an Improver or non‐Improver for each test data point according to the equation *P* (*C*_*i*_ | *x*) = (*P* (*C*_*i*_) * *p* (*x* | *C*_i_) / *p* (*x*) where *P*(*C*_*i*_) is the prior probability of class *C*_*i*_ (e.g., Improver) occurring, *p* (*x* | *C*_i_) is the conditional probability that class *C*_*i*_ is associated with feature observation *x*, and *p* (*x*) is the marginal probability that observation *x* is observed (effectively constant for any given dataset). The joint model (combining all features) can then be expressed as the product of the probabilities for all features, and the algorithm classifies unseen data as Improver or non‐Improver based on the highest probability.

### 2.7 Support vector machine

SVM classifiers find the maximum‐margin hyperplane using only those data instances closest to the separation boundary (i.e., “support vectors”) to determine classification boundaries (Burges, 1998). Both linear and nonlinear (using a kernel) classifications can be performed. Polykernel SVM classifiers were evaluated starting with an exponent of one and increasing in size until average accuracy (over all 1,000 allocations of test/training data) plateaued.

### 2.8 K star (K‐nearest neighbor)

The K* algorithm operates by assigning new data instances to the class that occurs most frequently amongst the *k*‐nearest data points, *y*_*j*_, where *j* = 1,2…*k* (Hart, 1968). Distance is then used to retrieve the most similar instances from the data set. The K* function is operationalized as K* (*y*_*i*_,*x*) = −ln p*(*y*_*i*_,*x*), where p* is the probability of all transformational paths from instance *x* to *y*, that is, the probability *x* will arrive at *y* via a random walk in feature space.

### 2.9 AdaBoost

AdaBoost operates by creating multiple weak classifiers that are weighed by their effectiveness at classifying data (Viola & Jones, 2001). Initially, a classifier is created with all instances weighted equally. Next, the weight of the incorrectly predicted instances is increased. The instances that are still misclassified are then selected and their weights increased as well, and so forth. After the complete classifier is constructed, each weak classifier then casts a weighted “vote” as to the class membership of each set of individual test data to make a classification decision.

### 2.10 J48 decision tree

Decision tree classifiers operate hierarchically, with each level representing a feature (e.g., left DLPFC activation) (Alpaydin, 2004; Quinlan, 1993). Based on the value of that feature the tree either classifies immediately or passes the information to the next level of the tree. The C4.5 algorithm (Quinlan, 1993) was used for the J48 decision tree, which uses a measure called “information gain” to select each attribute at each stage. In essence, the J48 tree first chooses the feature that most effectively splits the training data into one class or another using a measure called “information gain” (essentially, the effectiveness of feature at classifying data). After this split, the tree then chooses the next most effective feature to split each resulting partition. The process then iteratively repeats until all training data is classified. Performance of the resulting tree is then evaluated on test data.

### 2.11 Random forest

A random forest is a group of decision trees made up of random partitions of training data (Breiman, 2001). Each tree casts a “vote” as to the classification of a testing instance and votes are counted to produce the final classification.

### 2.12 Deep learning

DL was performed using a multi‐layer perceptron network trained using the ADAM optimizer in Pytorch (pytorch.org) to construct an architecture with parameters (e.g., weights) for optimal classification accuracy. The network consisted of multiple hidden layers (8–32–16–8) for the four‐ROI classifier and (252–128–64–8) for the within‐ROI voxelwise classifier with a single neuron as the output layer. After the network was learned, XAI methods were used to extract rules the deep learner used for classification (Davidson et al., 2018). These rules were in disjunctive normal form in that they consisted of alternative (disjunctions) of a combination (conjunction) of input neurons whose activations cooccur. This procedure was only performed for binary classifiers. Devising rules for continuous classifiers would require dividing the sample into quantiles. This procedure is likely to be underpowered given the already limited sample size of the study.

**FIGURE 1:**
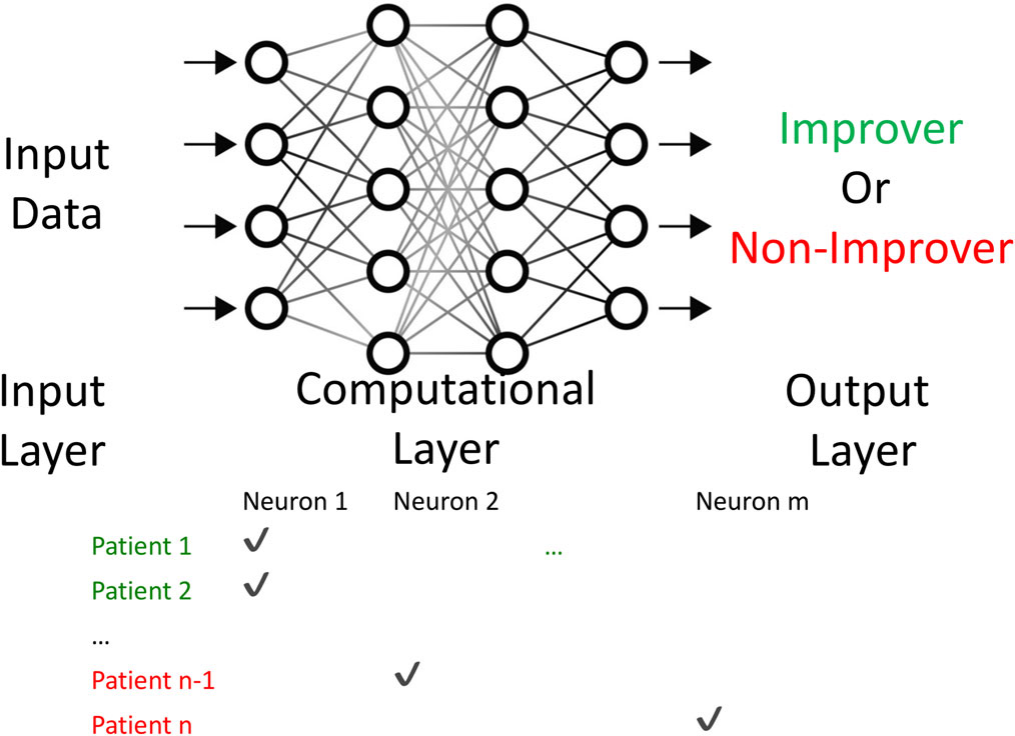
Example neural network for deep learning to illustrate explainable artificial intelligence procedures. The training data were divided into Improvers (green font, +) or non‐Improvers (red font, −). Each training instance*x*activated a subset of neurons which can be considered a simple binary vector (row). Using X^+^(Improvers) and X^−^(non‐Improvers) we set up a set‐style cover problem to find the subset of hidden nodes A^+^that activated for X^+^but that did not activate for X^−^, as well as the subset A^−^that activated for X^−^but not X^+^(see Section 2for more details)

Figure 1 illustrates how these explanations were generated. The training data were divided into Improvers or non‐Improvers (+,−). Each training instance *x* activated a subset of neurons which can be considered a simple binary vector (row). The collection of all neuron activations for each type of instance (+,−) was then simply two tables of input and hidden layer neuron activations X^+^ and X^−^ with the number of rows being the number of instances of each type and the number of columns being the number of neurons. Hence, the entry (*i*, *j*) of X^+^ was set to 1 if and only if Improver instance *i* activated node *j*. Using X^+^ and X^−^ we set up a set‐style cover problem to find the subset of hidden nodes (*A* ^+^) that activated for X^+^ but that did **not** activate for X^−^. Conversely, we found (*A* ^−^) which was a subset of hidden nodes that activated for X^−^ but not for X^+^. Importantly, *A* ^−^ and *A* ^+^ must not overlap on any hidden units. These two subsets were found using an integer linear programming optimization formulation as described in Davidson et al. (2018) and Sambaturu et al. (2020). Notably, XAI only outputs the most predictive rules (and not every rule), and therefore XAI outputs themselves may not perform as well as the entire deep learner.

### 2.13 Performance comparison

Accuracies were qualitatively examined by calculating mean accuracies (for the binary outcome) or mean root mean square errors (RMSEs, for the continuous outcome) and 95% confidence intervals for performance across the 1,000 random assortments of 90% training data and 10% test data for each algorithm. For the binary outcome, McNemar's test was also performed to determine if the proportion of average correct vs. incorrect predictions (i.e., average accuracies) were statistically significantly different (two‐tailed *p <* .05*)* between the DL algorithm and each other algorithm. As we were primarily interested in performance of the DL algorithm, McNemar's tests were only performed comparing the DL to each shallow learner or to the pooled (average) performance of all shallow learners.

## 3 RESULTS

### 3.1 Demographic and clinical

Demographic and clinical information (recapitulated from Smucny et al. (2019)) is presented in Tables 1 and 2. Mean BPRS score at baseline for all individuals with psychotic disorders was 42.7 (*SD* = 9.7). Mean BPRS score at follow‐up for the psychosis group was 37.3 (*SD* = 9.0). Then, 47% of BD and 60% of SZ participants showed greater than 20% decrease in total BPRS score (scaled to a lowest value of zero) at follow‐up and were classified as “Improvers”. Mean improvement in BPRS score for Improvers was 12.7 (*SD* = 7.3), corresponding to a 59% decrease.

**TABLE 1:** Demographic information. Numbers in parentheses represent the *SD*

	HC	Psychosis
*N*	138	82
Age	20.4 (2.7)	21.0 (3.2)
Gender (M/F)	85/53	59/23
AX‐1/AX‐2 protocol participants	73/65	52/30
Days to follow‐up	—	394.1 (138.5)

**TABLE 2:** Clinical information at baseline and follow‐up. Numbers in parentheses represent the *SD*

	Baseline	Follow‐up
Antipsychotics (Med/Unmed)	69/13	60/22
Antipsychotics (CPZ equivalent dose, mg/day)	227.4 (154.4)	307.3 (305.9)
BPRS improved/did not improve^a^	—	47/35 (57.3% improved)
Total BPRS score	42.7 (9.7)	37.3 (9.0)

Abbreviations: BPRS, Brief Psychiatric Rating Scale; CPZ, chlorpromazine; HC, healthy controls.

^a^ Clinical “improvement” was defined as showing >20% decrease (with lowest possible score [24] set to zero) on total BPRS score at follow‐up (vs. baseline). Only patients with total BPRS score >=29 at baseline were included in the sample.

As presented previously (Smucny et al., 2019), across individuals with psychosis and HCs, activation was observed in the DLPFC/SPC for both protocol versions (Supplementary Table 2, Supplementary Figure 1). Raw behavioral and fMRI ROI data segregated by protocol version are presented in Supplementary Table 3. Protocol‐adjusted beta weights for task‐associated activation for each ROI are presented in Supplementary Table 4.

### 3.2 Binary classifiers

Accuracy data for ML algorithms for the four ROI feature and voxelwise ROI feature classifiers are presented in Table 3. McNemar's test results are presented in Table 4. For SVM, accuracy peaked at an exponent of 3 for the polynomial kernel; accuracy using this kernel are hereafter reported. Qualitatively, the best overall performance was achieved using DL for both the four ROI feature and voxelwise ROI feature classifiers. Accuracies for the four‐ROI feature classifiers were only statistically significantly improved for DL versus the K* and Random Forest learners. Accuracies for the voxelwise ROI feature classifiers were significantly improved for DL vs. the majority of other algorithms as well as the pooled average of all shallow learners. The improvement in DL accuracy was driven by qualitative improvements in predicting both Improvers and non‐Improvers, suggesting performance changes were not exclusively driven by positive or negative predictive values. For four ROI feature classifiers, the J48 decision tree algorithm most closely approached the DL in regard to performance. Although the Naive Bayes learner showed identical accuracy to the J48 algorithm, its performance was driven by strong performance in non‐Improvers at the expense of performance in Improvers. For voxelwise ROI classifiers, the Naive Bayes learner most closely approached DL performance levels for overall accuracy, predicting Improvers, and predicting non‐Improvers.

**TABLE 3:** ML/DL performance for each method, using either mean BOLD signal within the four frontoparietal ROIs or voxelwise data within the four frontoparietal ROIs as features and Improver/non‐Improver status as a binary outcome. Numbers in parentheses represent the 95% CI over 1,000 repetitions of random 90% training/10% test data allocations. “Pooled” = average of all non‐DL methods

Method	ROI mean signal accuracy	ROI mean signal accuracy for Improvers	ROI mean signal accuracy for non‐Improvers	ROI voxelwise accuracy	ROI voxelwise accuracy for Improvers	ROI mean signal accuracy for non‐Improvers
Logistic regression	63.7% (62.8–64.7%)	53.1% (51.4–54.8%)	72.0% (70.8–73.2%)	62.4% (61.4–63.3%)	54.8% (53.1–56.4%)	68.2% (66.9–69.5%)
Naive Bayes	67.4% (66.5–68.4%)	56.5% (54.9–58.1%)	75.8% (74.6–77.0%)	68.6% (67.7–69.6%)	67.4% (65.9–68.9%)	69.6% (68.3–70.9%)
SVM (three kernel)	63.6% (62.6–64.5%)	57.7% (56.1–59.3%)	68.2% (66.9–69.6%)	56.8% (55.8–57.8%)	53.2% (51.6–54.9%)	59.6% (58.2–61.0%)
K*	60.8% (59.8–61.8%)	59.5% (57.9–61.1%)	61.9% (60.5–63.3%)	63.9% (62.9–65.0%)	63.5% (61.9–65.1%)	64.3% (62.9–65.7%)
AdaBoost	62.9% (61.9–63.9%)	61.1% (59.4–62.9%)	64.3% (63.0–65.6%)	60.9% (59.8–61.9%)	54.8% (53.2–56.5%)	65.5% (64.1–67.0%)
J48 decision tree	66.7% (65.7–67.7%)	69.3% (67.7–71.0%)	64.9% (63.5–66.2%)	54.8% (53.7–55.8%)	50.9% (49.2–52.6%)	57.7% (56.3–59.1%)
Random forest	63.4% (62.4–64.4%)	50.8% (49.2–52.5%)	72.8% (71.4–74.1%)	63.4% (62.4–64.4%)	56.2% (54.6–57.8%)	69.0% (67.7–70.3%)
Pooled (non‐DL methods)	64.1% (63.7–64.5%)	58.3% (57.7–58.9%)	68.5% (68.0–69.1%)	61.5% (61.1–61.9%)	57.3% (56.6–57.9%)	64.9% (64.3–65.4%)
DL	70.0% (69.8–70.2%)	65.9% (65.7–66.1%)	74.9% (74.8–75.1%)	72.6% (72.0–73.2%)	78.4% (77.1–79.6%)	70.2% (69.4–71.1%)

Abbreviations: BOLD, blood oxygenation level dependent; CI, confidence interval; DL, deep learning; ML, machine learning; SVM, support vector machine.

**TABLE 4:** Statistical comparison (McNemar's test) of average accuracies (over the 1,000 repetitions) for the DL versus other methods, using either mean BOLD signal within the four frontoparietal ROIs or voxelwise data within the four frontoparietal ROIs as features and Improver/non‐Improver status as a binary outcome. “Pooled” = average of all non‐DL methods

Method	ROI mean signal *p* vs. DL accuracy	ROI mean signal *p* vs. DL accuracy for Improvers	ROI mean signal *p* vs. DL accuracy for non‐Improvers	ROI Voxelwise *p* vs. DL accuracy	ROI Voxelwise *p* vs. DL accuracy for Improvers	ROI Voxelwise *p* vs. DL accuracy for non‐Improvers
Logistic regression	0.063	0.031	1.000	0.004	0.001	1.000
Naive Bayes	0.500	0.125	1.000	0.125	0.063	1.000
SVM (3 kernel)	0.063	0.125	0.500	<0.001	<0.001	0.125
K*	0.016	0.250	0.125	0.008	0.016	0.500
AdaBoost	0.063	0.500	0.250	0.002	0.001	0.500
J48 decision tree	0.250	0.500	0.250	<0.001	<0.001	0.063
Random forest	0.031	0.016	1.000	0.008	0.001	1.000
Pooled (non‐DL methods) vs. DL	0.125	0.125	0.500	0.002	0.002	0.500

Abbreviations: BOLD, blood oxygenation level dependent; DL, deep learning; SVM, support vector machine.

For the four ROI feature classifier, XAI revealed the most predictive feature used by the deep learner was left DLPFC activation. Specifically, the most predictive rule was left DLPFC adjusted beta > .017 → Improver, left DLPFC adjusted beta ≤.017 → non‐Improver. This classification threshold (adjusted beta = .017) was intermediate to the HC mean (adjusted beta = .15, 95% CI = −0.02 to 0.31) and psychosis group mean (adjusted beta −.13, 95% CI = −0.37 to 0.11).

### 3.3 Continuous classifiers

RMSE data for ML algorithms for the four ROI feature and voxelwise ROI feature continuous outcome classifiers are presented in Table 5. In contrast to the binary outcome classifiers above (Improvement/non‐Improvement), these classifiers predicted change in total BPRS score from baseline to follow‐up.

**TABLE 5:** RMSEs and 95% CIs for using either mean BOLD signal within the four frontoparietal ROIs or voxelwise data within the four frontoparietal ROIs as features and change in BPRS score as a continuous outcome. Classification for algorithms that require nominal outcomes (Bayes, AdaBoost, J48, random forest) was performed by dividing BPRS scores into 10 bins (see Methods). Numbers in parentheses represent the 95% confidence interval over 1,000 repetitions of random 90% training/10% test data allocations

Method	ROI mean signal RMSE (95% CI)	∆ from DL (95% CI)	ROI voxelwise RMSE (95% CI)	∆ from DL (95% CI)
Linear regression	10.41 (10.24–10.57)	9.9% (8.6–11.0%)	10.94 (10.78–11.11)	37.1% (35.9–38.4%)
Naive Bayes	10.94 (10.77–11.11)	15.5% (14.2–16.7%)	14.11 (13.91–14.32)	76.8% (75.4–78.3%)
SVM (1 kernel)	10.30 (10.14–10.46)	8.8% (7.5–9.9%)	13.33 (13.11–13.55)	67.0% (65.3–68.7%)
K*	11.10 (10.93–11.28)	17.2% (15.9–18.5%)	12.39 (12.21–12.58)	55.3% (54.0–56.7%)
AdaBoost	10.43 (10.26–10.61)	10.1% (8.8–11.4%)	10.95 (10.78–11.11)	37.2% (35.9–38.4%)
J48 decision tree	12.23 (12.04–12.42)	29.1% (27.7–30.5%)	13.41 (13.21–13.61)	68.0% (66.6–69.5%)
Random forest	10.43 (10.26–10.60)	10.1% (8.8–11.3%)	10.48 (10.31–10.66)	31.3% (30.0–32.8%)
Pooled (non‐DL methods)	10.84 (10.77–10.90)	14.5% (14.2–14.5%)	12.23 (12.15–12.31)	53.3% (53.2–53.3%)
DL	9.47 (9.43–9.52)	—	7.98 (7.93–8.03)	—

Abbreviations: BOLD, blood oxygenation level dependent; CI, confidence interval; DL, deep learning; RMSE, root mean square error; SVM, support vector machine.

As demonstrated for the binary classifiers, DL outperformed all shallow ML algorithms as well as the pooled average of the ML algorithms as demonstrated by qualitatively lower RMSE values (Table 5). The DL performance differences were magnified for the voxelwise ROI features.

## 4 CONCLUSIONS

The results of this study suggest that DL performs better than shallow ML algorithms in regard to predicting BPRS improvement in recent onset psychosis using cognitive control‐associated activations from frontoparietal brain regions, supporting the development of DL‐based methods for predicting treatment outcomes in psychosis populations. The performance enhancement in DL was not specific to either Improvers on non‐Improvers, suggesting positive predictive value was not enhanced at the expense of negative predictive value (or vice versa). XAI revealed that the most predictive feature used by the binary DL was left DLPFC activation. Overall, our study therefore illustrates the utility DL in combination with XAI to maximize predictive power while also shedding light on how to solve the “black box” problem inherent in most ML algorithms by revealing the bases for DL‐based prediction. The finding that DL was the best method at predicting the continuous outcome (change in BPRS score) that the predictive enhancement observed with DL is nonspecific to any particular definition of 1‐year clinical “Improvement” in psychosis.

Improved performance with DL is consistent with prior research comparing DL with shallow architectures (Krizhevsky et al., 2012; Le et al., 2012). Although the reason(s) for this improvement have not been experimentally verified, it is likely related to the ability of DL to combinatorically use input features to improve prediction. This effect may be magnified as features are added to classifier, as evidenced by larger performance differences between DL and shallow architectures using the voxelwise ROI feature sets. It should also be noted that for the four ROI feature binary outcome, although XAI methods found that the most important classification rule was a simple threshold for one feature, additional, more complex combinations of features may have also been utilized by the deep learner to refine its predictions that were not individually predictive enough to be identified by XAI.

Although our study was limited by its sample size (and therefore had limited power to detect statistically significant differences, as evidenced by the mostly nonsignificant McNemar's tests), we purposefully used the same sample as our previous report (Smucny et al., 2019) in order to directly compare accuracies with the prior logistic regression‐based approach. As hypothesized, the deep learner outperformed the logit function. As our study was conducted at a single site with a small sample size, however, future studies using more participants and/or multiple sites will be necessary to determine the generalizability of our findings. It is nonetheless notable that such high accuracy (70%) toward predicting outcome could be achieved using brain activations from a single, simple cognitive‐control task. It would be interesting to see if additional features (e.g., striatal dopamine levels, as previously demonstrated for differentiating treatment resistant vs. responsive patients with SZ (Demjaha, Murray, McGuire, Kapur, & Howes, 2012)) could be used in future large‐scale studies to further improve outcome prediction toward a clinically implementable level (i.e., at least 80% accuracy) while guarding against overfitting. Our results suggest that deep learner may be the most powerful tool to utilize for this purpose.

Another limitation to consider when interpreting our results was the naturalistic nature of our prospective study. Specifically, we did not use strict guidelines on the use of antipsychotic medications across time, and medication compliance was ascertained by self‐report. It is unclear, therefore, if symptomatic change in this study was due to medications or other forms of treatment (e.g., psychotherapy, psychoeducation). For this reason, in this study and our previous work using this sample, we labeled participants with psychosis as “Improvers” and “non‐Improvers” rather than responders/nonresponders. On the other hand, the application of this approach in a naturalistic setting where personalized evidenced‐based practices are provided does speak to the potential generalizability of this approach and utility for future clinical applications.

Finally, although we argue that 80% accuracy is desirable to achieve clinical utility, others have argued that the effectiveness of a prognostic indicator should be solely based on their ability to change clinical practice (e.g., Perlis, 2011). It is possible, therefore, that the 73% accuracy achieved in this study with DL is sufficient to suggest using fMRI scanning during the described cognitive control task to predict clinical improvement in psychosis. Nonetheless, as neuroimaging studies sometimes have questionable reproducibility (Elliott et al., 2020), our results require replication using a larger sample before strong statements can be made regarding our method's clinical utility (Perlis, 2011). To our knowledge, however, no established method has been developed that can reliably predict long‐term symptomatic improvement in early psychosis patients. In combination with our prior work using logistic regression, we believe that the present study represents an important preliminary step toward developing a predictive algorithm for this purpose.

## CONFLICT OF INTEREST

Dr I. D. has an active research grant from Google and a previous grant from Yahoo!.

## Supporting information


**Appendix** **S1**: Supporting information

## Data Availability

The data that support the findings of this study are available from the corresponding author upon reasonable request.
