# Supplementary material for: Comparing machine and deep learning‐based algorithms for prediction of clinical improvement in psychosis with functional magnetic resonance imaging
Source: Hum Brain Mapp. 2020 Nov 13;42(4):1197–205. doi: 10.1002/hbm.25286 (PMC7856652; doi:10.1002/hbm.25286)
Supplement: Supplementary file 1 — Appendix S1: Supporting information [file HBM-42-1197-s001.docx]

| **Protocol** | **%AX/AY/BX/BY Trials** | **Total Trials** | **Cue Duration (ms)** | **Probe Duration (ms)** | **ISI (ms)** | **ITI (ms)** | **Total Time** |
| --- | --- | --- | --- | --- | --- | --- | --- |
| **AX-1** | 70/10/12.5/7.5 | 4x40 = 160 | 500 | 500 | 3500 | 9500 | 37m 20s |
| **AX-2** | 60/10/15/15 | 5x40 = 200 | 500 | 500 | 2200-3500 (jittered) | 1700-14500 (jittered) | 30m 20s |

**Supplementary Table 1a.** Task parameters for AX-CPT Protocols 1 (AX-1) and 2 (AX-2). Abbreviations: ISI = Interstimulus Interval, ITI = Intertrial Interval.

**Supplementary Table 1b.** Scanning parameters for AX-CPT Protocols 1 (AX-1) and 2 (AX-2).

| **Protocol** | **Scanner** | **TR (ms)** | **TE (ms)** | **Flip Angle** | **FOV (cm)** | **# Slices** | **Resolution (mm)** | **Acquisition** |
| --- | --- | --- | --- | --- | --- | --- | --- | --- |
| **AX-1** | 1.5T Signa (GE Healthcare) | 2000 | 40 | 90**°** | 22x22 | 24 | 3.40x3.40x 4.00 | Contiguous, Interleaved |
| **AX-2** | 3.0T Tim Trio (Siemens) | 2000 | 29 | 77**°** | 24x24 | 32 | 3.75x3.75x3.5 | Contiguous, Interleaved |

**Supplementary Table 2.** Whole-brain results showing significant (height *p* < 0.001, whole-brain cluster *p_FDR_* <0.05) activation for the B > A contrast (correct trials only) in expected brain regions for each protocol version (AX-1 and AX-2) across all participants.^[[1]](#footnote-1)^

| **Protocol Version** | **Brain Region** | **Hemi** | **Cluster *p*_FDR_** | **Cluster Size (Voxels) at Voxelwise *p*< 0.001** | **Peak x,y,z** | **Peak *p*** | **Peak t** |
| --- | --- | --- | --- | --- | --- | --- | --- |
| AX-1 | DLPFC | L | <0.001 | 1909 | -48, 8, 34 | <0.001 | 6.93 |
| AX-1 | SPC | R | <0.001 | 2364 | 34, -68, 52 | <0.001 | 6.13 |
| AX-1 | DLPFC | R | <0.001 | 1085 | 54, 12, 36 | <0.001 | 5.84 |
| AX-1 | SPC | L | <0.001 | 1716 | -28, -72, 46 | <0.001 | 5.36 |
| AX-2 | DLPFC | R | <0.001 | 69931 (contiguous cluster)^[[2]](#footnote-2)^ | 52, 14, 38 | <0.001 | 9.16 |
| AX-2 | SPC | R | <0.001 |  | 54, -34, 52 | <0.001 | 10.01 |
| AX-2 | DLPFC | L | <0.001 |  | -50, 8, 26 | <0.001 | 7.51 |
| AX-2 | SPC | L | <0.001 |  | -52, -38, 50 | <0.001 | 8.05 |

**Supplementary Table 3.** Raw behavioral and functional data segregated by protocol version. Numbers in parentheses represent the standard deviation.

| **Measure** | **AX-1** | **AX-2** |
| --- | --- | --- |
| **D-Prime Context** | 3.31 (0.83) | 2.86 (0.79) |
| **Left DLPFC ROI BOLD (B > A Cue)** | 0.27 (1.06) | 0.55 (1.09) |
| **Right DLPFC ROI BOLD (B > A Cue)** | 0.21 (1.15) | 0.57 (1.02) |
| **Left SPC ROI BOLD (B > A Cue)** | 0.53 (1.15) | 0.95 (1.93) |
| **Right SPC ROI BOLD (B > A Cue)** | 0.57 (1.11) | 0.99 (1.35) |
| **Accuracy (%)** | | |
| AX Trials | 96.08 (6.02) | 92.10 (7.03) |
| AY Trials | 82.31 (19.20) | 79.18 (16.55) |
| BX Trials | 89.61 (12.45) | 88.66 (11.08) |
| BY Trials | 97.71 (5.54) | 96.08 (6.98) |
| **Reaction Time** | | |
| AX Trials | 576.38 (140.39) | 460.61 (77.12) |
| AY Trials | 747.37 (150.69) | 577.64 (90.11) |
| BX Trials | 678.35 (235.04) | 471.05 (125.46) |
| BY Trials | 607.76 (173.64) | 470.77 (103.82) |

**Supplementary Table 4.** Protocol-adjusted beta weights for each ROI for each group (healthy controls (HC) and individuals with psychosis). Numbers in parentheses represent the standard deviation.

| **Measure** | **HC** | **Psychosis** |
| --- | --- | --- |
| **Left DLPFC ROI BOLD (B > A Cue)** | 0.15 (0.96) | -0.13 (1.09) |
| **Right DLPFC ROI BOLD (B > A Cue)** | 0.17 (0.99) | -0.16 (0.90) |
| **Left SPC ROI BOLD (B > A Cue)** | 0.22 (0.93) | -0.16 (1.05) |
| **Right SPC ROI BOLD (B > A Cue)** | 0.21 (0.98) | -0.11 (0.95) |


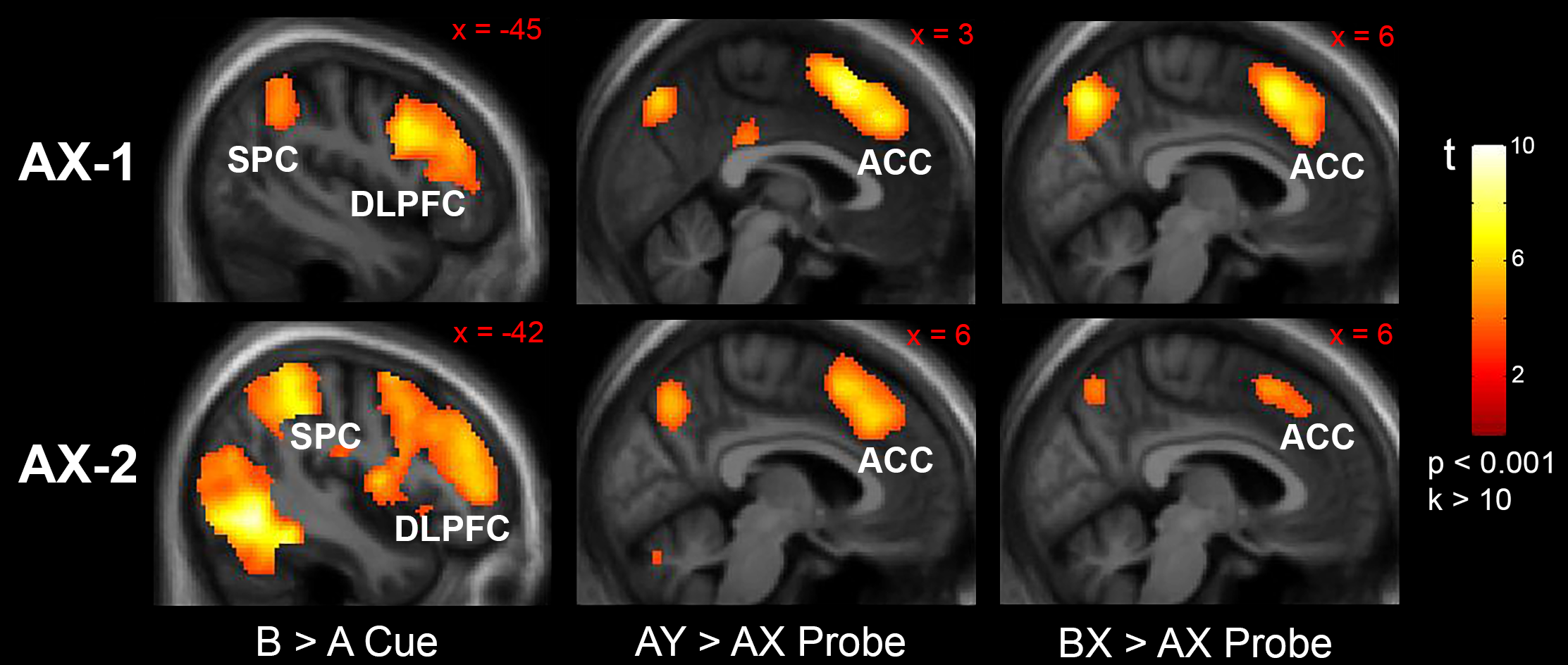
**Supplementary Figure 1.** Statistical parametric maps of the proactive control (B > A Cue) contrast for each AX protocol version (AX-1 and AX-2) across all participants. Significant activation was observed in the superior parietal cortex (SPC) and dorsolateral prefrontal cortex (DLPFC) in each protocol version (see Supplementary Table 2 for peak p values, peak t values, and cluster sizes at threshold). Maps thresholded at voxelwise p < 0.001, cluster size k > 10 voxels and masked with an inclusive gray matter mask for visualization.

1. Abbreviations: ACC = Anterior Cingulate Cortex, DLPFC = Dorsolateral Prefrontal Cortex, SPC = Superior Parietal Cortex. [↑](#footnote-ref-1)
2. Left/Right SPC and DLPFC break into separate clusters at higher significance thresholds. [↑](#footnote-ref-2)
